# Analysis of Population Structure and Selection Signatures in Two Iraqi Fat‐Tailed Sheep Breeds

**DOI:** 10.1002/vms3.70584

**Published:** 2025-08-26

**Authors:** Mervan Bayraktar, Omer Shoshin, K. A. Saravanan, Tahreer M. Al‐Thuwaini, Gökhan Gökçe, Zeynab Hussein Fadhil, Melis Çelik Güney

**Affiliations:** ^1^ Department of Animal Science, Faculty of Agriculture Çukurova University Adana Turkey; ^2^ College of Veterinary, Physiology Science University of Kirkuk Kirkuk Iraq; ^3^ Animal Genetics and Breeding ICAR‐Central Sheep and Wool Research Institute (ICAR‐CSWRI) Avikanagar India; ^4^ Department of Animal Production, College of Agriculture Al‐Qasim Green University Al Qasim Babil Iraq; ^5^ Department of Biology, College of Science University of Kirkuk Kirkuk Iraq

**Keywords:** Awassi, Hamdani, integrated haplotype score (iHS), population structure, runs of homozygosity (ROH), selection signatures, Tajima's D

## Abstract

This study aimed to elucidate the genetic architecture and selection signatures in two prominent Iraqi fat‐tailed sheep breeds, Awassi and Hamdani. One hundred forty animals were genotyped using the Illumina Ovine SNP50K BeadChip, providing genome‐wide coverage with approximately 53,714 SNP markers. To uncover candidate genomic regions under selection, we employed three complementary approaches: integrated haplotype score (iHS), runs of homozygosity (ROH) and Tajima's D analyses. The iHS analysis identified 69 candidate genes in Awassi sheep and 286 in Hamdani sheep, whereas ROH analysis revealed 190 and 277 candidate genes in the respective breeds. Tajima's D corroborated these findings with one gene in Awassi and four in Hamdani sheep. Population structure was rigorously assessed using ADMIXTURE, principal component analysis (PCA) and neighbour‐joining phylogenetic tree reconstruction, collectively demonstrating a distinct genetic separation of the Awassi breed and a more admixed genetic profile for the Hamdani breed. Functional enrichment analysis of candidate genes implicated several biological processes and pathways, including immune response, hormone regulation and cellular signalling, underscoring their potential roles in adaptation and disease resistance. Overall, our findings provide novel insights into the genetic differentiation and adaptive evolution of Iraqi fat‐tailed sheep, offering a valuable resource for future breeding and conservation programmes.

## Graphical abstract

Awassi sheep exhibit clear genetic separation, whereas Hamdani sheep show an admixed profile, with iHS, ROH and Tajima's D analyses identifying breed‐specific candidate genes. Enrichment in immune response, hormone regulation and cellular signalling pathways highlights adaptive evolution and guides future breeding and conservation.

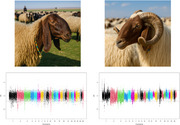

## 1 Introduction

Sheep farming is a cornerstone of agricultural systems in the Middle East, playing significant economic, social and cultural roles. In Iraq, sheep represent a vital livestock resource, with an estimated population of approximately 8 million animals distributed across diverse ecological zones (Jaradat 2003; Zeder 2008, 2011; Al‐Jabar and Al‐Thuwaini 2024). Iraqi sheep breeds have evolved unique adaptive traits that enable them to thrive in semi‐arid and arid environments characterized by low annual precipitation (200–500 mm), limited natural pastures and pronounced daily and seasonal temperature fluctuations. These adaptations include endurance for long‐distance grazing, tolerance to extreme temperature variations, drought resilience and an enhanced ability to utilize sparse vegetation. Despite these remarkable adaptations, the overall productivity in terms of meat, milk, wool and reproductive efficiency remains modest, thus constraining the full economic potential of sheep production in the region (Bayraktar and Shoshin 2022). Among the indigenous breeds, Awassi and Hamdani sheep are two of the most economically significant and widely reared in Iraq. The Awassi, originally associated with the Syrian Desert (Badia Al‐Sham), is extensively distributed across Iraq, Syria, Jordan, Turkey, Palestine and Lebanon and comprises approximately 60% of Iraq's sheep population. This breed is characterized by a medium body size, moderate fertility rates, satisfactory milk production and a distinctive fat‐tailed morphology—an essential adaptation for energy storage in harsh environments (Galal et al. 2008; Al‐Samarai et al. 2016; Mohammed 2022; Meydan et al. 2024; Aljubouri et al. 2025). In contrast, the Hamdani breed, predominantly reared in Northern Iraq, is recognized for its larger body size, high milk yield, superior lamb growth performance, increased twinning rates and unique morphological features, such as significantly pendulous ears, which local farmers associate with enhanced productivity. Although both breeds exhibit beneficial adaptations to local environmental pressures, their genetic potential for economically essential traits—including growth rate, milk yield, reproductive performance and wool characteristics—remains insufficiently explored at the molecular level (Bingöl and Bingöl 2018; Bayraktar and Shoshin 2021; Güzel et al. 2022). Previous genetic studies have shown that domesticated sheep breeds often exhibit complex population structures resulting from geographic isolation, distinct ecological pressures, human‐mediated selection and interbreeding among breeds. In Iraq, limited geographic barriers, traditional livestock management practices and seasonal migration patterns have fostered extensive genetic admixture among indigenous breeds, complicating efforts to characterize and conserve the Awassi and Hamdani populations genetically. Moreover, current breeding practices primarily rely on traditional selection criteria based on observable phenotypic traits rather than underlying genetic merit, which can potentially reduce genetic diversity and undermine the sustainable use of these valuable genetic resources (Alnajm 2020; Fadhil and Al‐Shuhaib 2022; Mohammed 2009). Recent advances in genomic technologies—particularly high‐density SNP genotyping arrays and modern bioinformatics pipelines—offer powerful tools for analysing population structure, assessing genetic diversity and identifying signatures of selection in livestock populations (Hayes et al. 2009; Gutierrez‐Reinoso et al. 2021). Detecting genomic regions under selection can reveal genes and biological pathways that contribute to adaptive and economically relevant traits, providing critical guidance for targeted breeding programmes, enhanced productivity, preservation of genetic diversity and sustainable livestock production systems (Vitti et al. 2013; Qanbari and Simianer 2014; Karacaören 2020; Karsli et al. 2024; Karabaş and Yılmaz 2025). Complementary statistics are widely used to uncover these selection footprints: The integrated haplotype score (iHS) quantifies the decay of extended‐haplotype homozygosity around ancestral versus derived alleles and is highly sensitive to incomplete or ongoing selective sweeps over the last ∼30–50 generations (Voight et al. 2006; Gautier and Vitalis 2012); runs of homozygosity (ROH) detect long, uninterrupted tracts of homozygous genotypes whose excess indicates either historical positive selection driving a favourable haplotype toward fixation or very recent inbreeding (McQuillan et al. 2008; Biscarini et al. 2019); and Tajima's D contrasts average pairwise nucleotide diversity with the total number of segregating sites, with significant positive or negative deviations pointing to balancing or directional selection, respectively (Tajima 1989; Sabeti et al. 2002). By integrating haplotype‐, homozygosity‐ and frequency‐based metrics, these methods collectively capture both recent and ancient selective events across different allele‐frequency spectra. Despite the importance of Awassi and Hamdani sheep in Iraq, comprehensive genome‐wide studies of their population structure, genetic diversity and selection signatures are lacking. Therefore, this study provides a detailed characterization of genetic variation and differentiation within and between these two breeds by applying these state‐of‐the‐art genomic tools to pinpoint specific loci under selection.

## 2 Materials and Methods

### 2.1 Populations and Sample Collection

**FIGURE 1:**
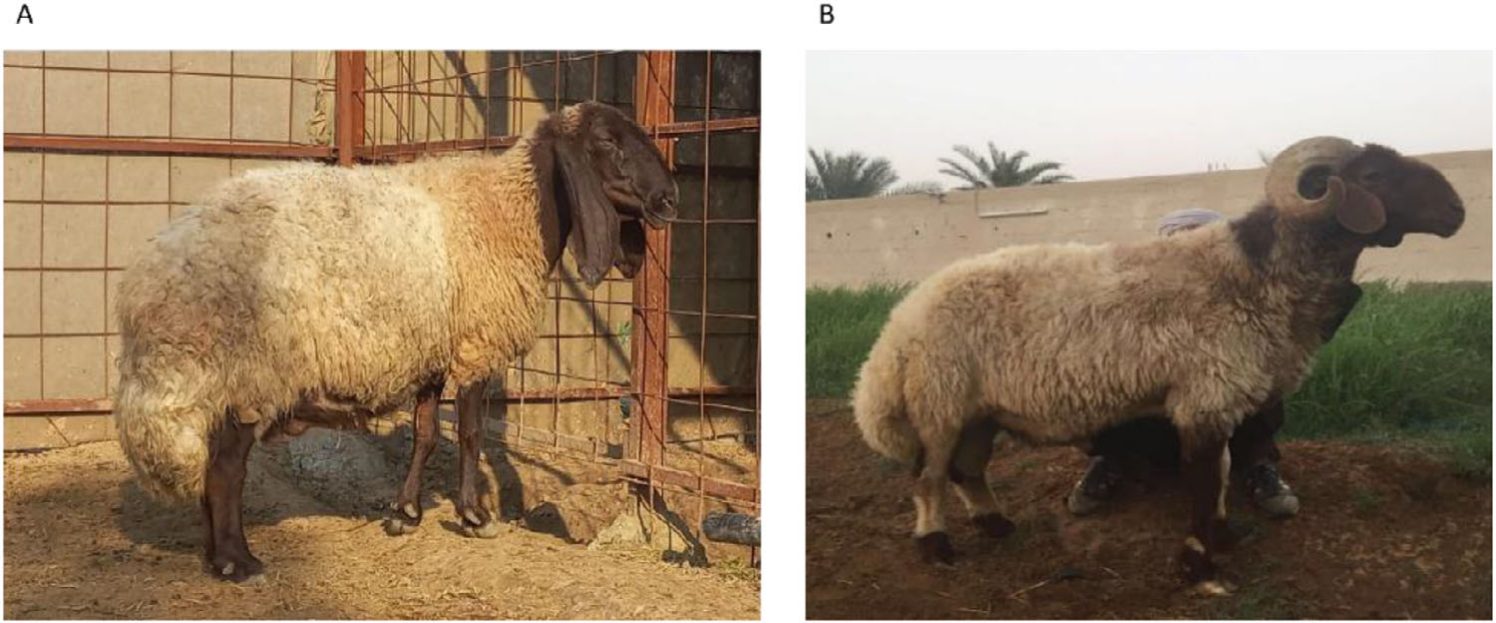
(A) Hamdani breed and (B) Awassi breed.

A total of 140 blood samples, comprising 70 Awassi sheep and 70 Hamdani sheep (Figure 1), were collected from two independent private farms in Kirkuk City, Iraq. The Awassi sheep were obtained from Farm A, situated south of Kirkuk City, whereas the Hamdani sheep originated from Farm B, located north of Kirkuk City. Both farms employ similar semi‐intensive management and feeding regimes. From each farm's eligible population, 70 animals were then randomly chosen to minimize relatedness bias. Blood samples (10 mL) were collected via jugular venipuncture into EDTA tubes, stored on ice and transported to our laboratory for genomic DNA extraction. A licensed veterinarian collected the blood samples, and the ethics committee approved the sampling protocol (Ethics Report KIR2024‐658).

### 2.2 DNA Extraction and Genotyping

Genomic DNA was extracted from the blood samples using the chloroform extraction method. The quality and concentration of the extracted DNA were confirmed before proceeding with genotyping. The animals were genotyped using the Illumina Ovine SNP50K BeadChip, which assays approximately 53,714 SNPs across the sheep genome, with positions referenced to the sheep reference genome Oar_v4.0.

### 2.3 Genotype Quality Control

Quality control of the genotype data was performed using PLINK (Purcell et al. 2007). SNPs were filtered by excluding those with a genotype missing rate above 10%, those that deviated from the Hardy–Weinberg equilibrium at a significance level of *p* < 0.0001, and those with a minor allele frequency of less than 5%.

### 2.4 Genetic Diversity and Population Structure Analysis

#### 2.4.1 Principal Component Analysis (PCA)

PCA was conducted to explore the population structure and genetic differentiation between the two breeds. The SNP dataset was first normalized, and PCA was performed using the prcomp() function in R (R Core Team 2024). The first two principal components (PCs) were extracted and visualized using the ggplot2 package (Wickham and Sievert 2009), with individuals colour‐coded according to their breed. The variance explained by PC1 and PC2 was adjusted to 70% and 17%, respectively, to optimize the visualization of population differentiation.

### 2.5 Admixture Analysis

Admixture analysis was performed using the LEA package (Frichot and François 2015) in R to estimate the ancestry proportions of individuals from Awassi and Hamdani sheep. The snmf() function was used to compute ancestry coefficients for various values of K (the number of ancestral clusters). A cross‐validation (CV) error analysis was performed, ranging from *K* = 1 to *K* = 5, to determine the most suitable number of clusters. The lowest CV error was observed at *K* = 3, indicating the presence of three ancestral genetic components in the dataset. The admixture proportions were visualized using a stacked bar plot generated with ggplot2 (Wickham and Sievert 2009).

### 2.6 Phylogenetic Tree Construction

A phylogenetic tree was constructed to investigate the genetic relationships between individuals further. First, an Euclidean distance matrix was computed using the first three PCs obtained from PCA. A neighbour‐joining (NJ) tree was then constructed using the nj() function from the ape package (Paradis and Schliep 2019). The phylogenetic tree was visualized in a circular format using the ggtree package (Yu et al. 2017), with Awassi and Hamdani individuals colour‐coded for better interpretation.

### 2.7 Selection Signatures Analysis

Selection signatures were investigated using three complementary approaches: the iHS, ROH and Tajima's D. For the iHS analysis, haplotype phasing was performed using Beagle (Browning et al. 2018), and iHS values were calculated using the rehh package (Gautier and Vitalis 2012). Before applying any significance threshold, the top 5% of SNPs, ranked by absolute iHS score, were selected for downstream analysis. Candidate regions showing strong evidence of recent positive selection were identified by applying a threshold for iHS within 100‐kb sliding windows. Standardized iHS values and corresponding −log10(*p*) statistics were derived for all markers across the genome. Candidate selection regions were identified by scanning non‐overlapping 100 kb windows and marking any region containing at least one SNP with a −log10(*p*) value of 4 or greater (i.e., *p* < 10^4^). Windows meeting this criterion were designated as candidate regions, and these intervals were consolidated into a final list for downstream interpretation. The ROH analysis was performed using the detectRUNS package (Biscarini et al. 2019), employing a sliding window of 15 SNPs, a minimum requirement of 15 SNPs per run and a threshold for the proportion of overlapping homozygous windows. Additional parameters included a maximum gap between consecutive SNPs of 1 Mb, a minimum run length of 1,000,000 bp and a minimum SNP density of one SNP per 100 bp. Tajima's D was calculated using VCFtools (Danecek et al. 2011) with a sliding window size of 10,000 bp to identify genomic regions potentially under balancing selection. To identify genes overlapping with the candidate regions from iHS, ROH and Tajima's D analyses, we used a consistent annotation pipeline implemented in R. The biomaRt package was used to query the Ensembl database for the sheep reference genome Oar_v3.1. Candidate regions from each method were extended by 100 kb upstream and downstream to account for potential regulatory elements, creating regions of interest (RoI_100 kb). These regions were formatted using the tidyverse package. The biomaRt query retrieved Ensembl gene IDs, gene names and descriptions for genes that overlap with these regions, requiring at least one base pair of overlap. Overlapping regions across the three methods were visualized using a Venn diagram generated with the online tool Venny (Oliveros 2016).

### 2.8 GO and KEGG Functional Enrichment Analysis

Candidate gene lists from the iHS, ROH and Tajima's D analyses were annotated using the biomaRt package with the Oar_v3.1 sheep reference genome. Candidate regions from each method were extended by 100 kb upstream and downstream to account for potential regulatory elements, creating areas of interest (RoI_100 kb). These regions were formatted using the tidyverse package. The biomaRt query retrieved Ensembl gene IDs, gene names and descriptions for genes that overlap with these regions, requiring at least one base pair of overlap. Functional enrichment analysis of candidate genes was performed using g: Profiler via the gprofiler2 R package (Raudvere et al. 2019; Kolberg et al. 2020). We specified the organism as ‘ovaries’ and used Ensembl Oar_v3.1 as the reference annotation. Queries included GO: BP, GO: MF, GO: CC and KEGG sources, with multiple‐testing correction by FDR and a significance threshold of *q* < 0.05. Results (term IDs, names, sources, *p* values, *q* values, intersection sizes) were exported to an Excel file for record‐keeping using the writexl package. For graphical presentation, we used SRplot (Tang et al. 2023), an online platform for data visualization. The top 10 enriched GO and KEGG terms (ranked by *q* value) were plotted as barplots (−log_10_ *p* value) and dotplots (−log_10_ *p* value vs. intersection size), facilitating a clear comparison of functional categories under selection in Awassi and Hamdani sheep.

## 3 Results

### 3.1 Population Genetic Structure

**FIGURE 2:**
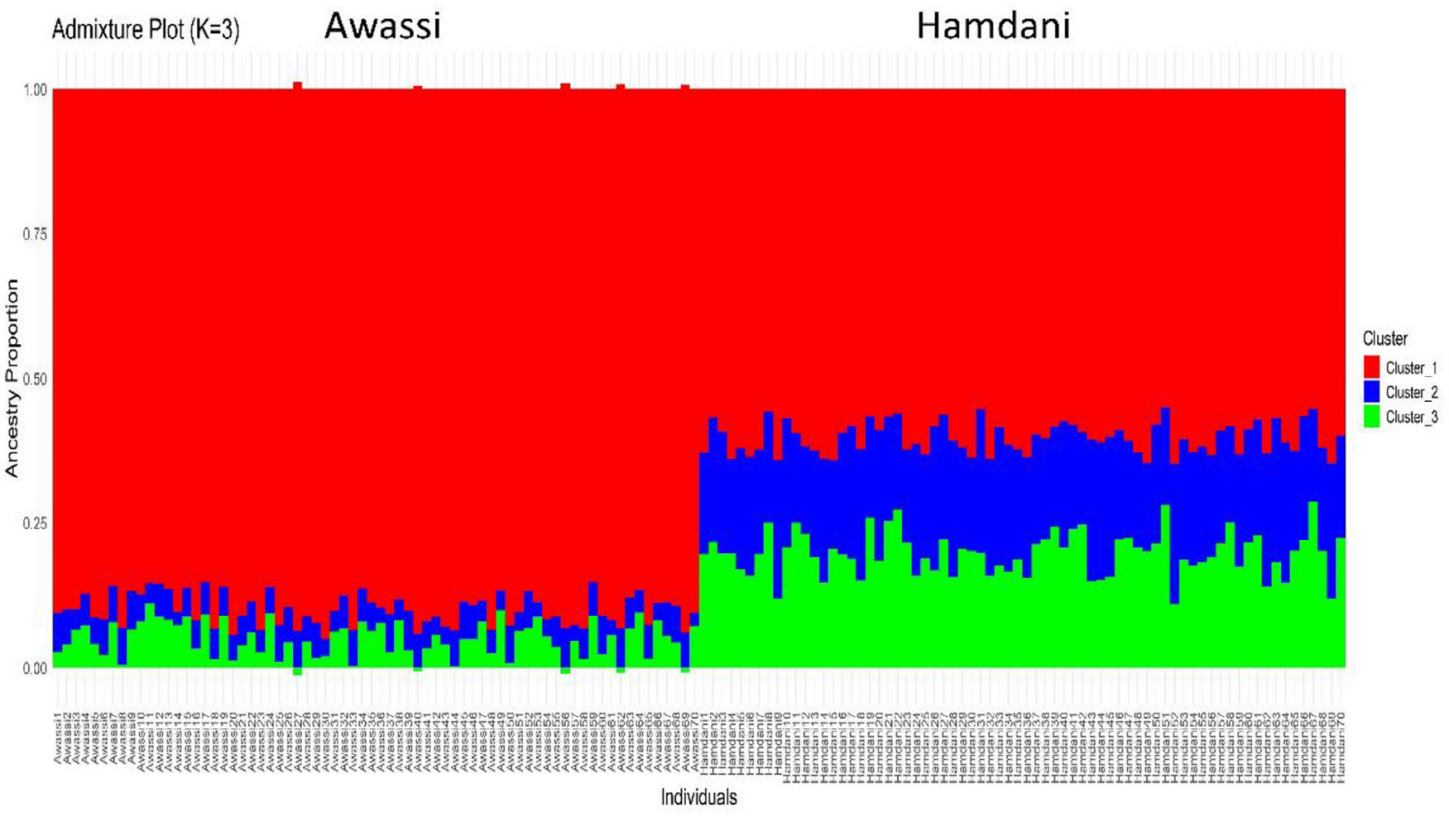
Admixture plot (*K* = 3) illustrating ancestral proportions in Awassi and Hamdani sheep.

**FIGURE 3:**
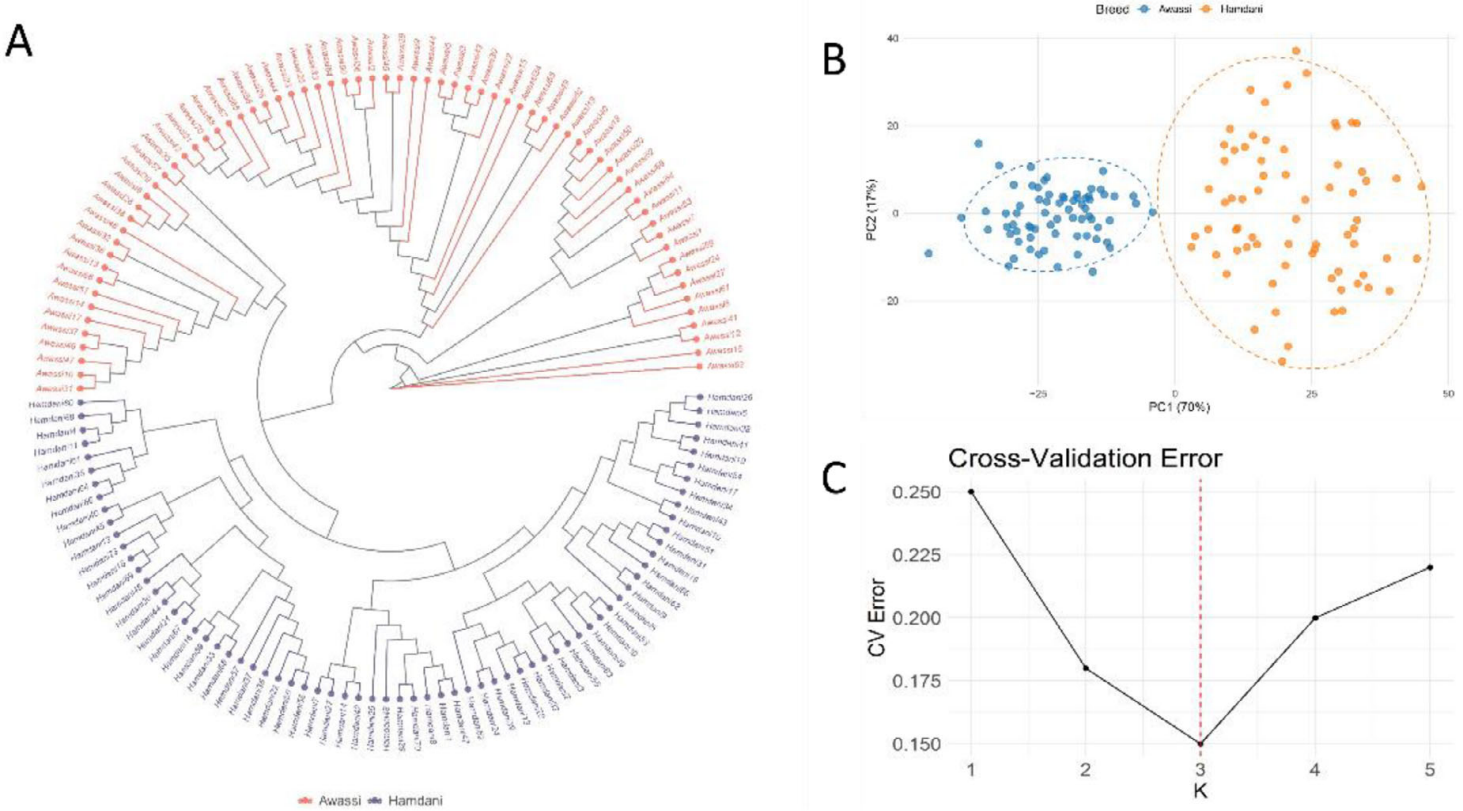
Population structure analysis of Awassi and Hamdani sheep: (A) neighbour‐joining tree of Awassi and Hamdani sheep, (B) PCA plot and (C) cross‐validation error for different *K* values in the admixture analysis.

Population genetic structure analysis using ADMIXTURE revealed that the optimal number of genetic clusters was three (*K* = 3) (Figure 2). At *K* = 3, the Awassi sheep predominantly formed a single, distinct genetic group, displaying a high membership proportion in Cluster 1, ranging from approximately 85% to 95%. This indicates clear genetic differentiation between the Awassi and Hamdani sheep breeds. In contrast, Hamdani individuals exhibited considerable admixture among the three identified clusters, suggesting a more heterogeneous genetic structure within this breed. The PCA further supported these findings, with the first two PCs explaining approximately 70% (PC1) and 17% (PC2) of the genetic variance. Awassi sheep individuals formed a cohesive cluster, predominantly located on the negative side of PC1. In contrast, the Hamdani sheep were distributed mainly positively, highlighting the genetic divergence between the two breeds. Phylogenetic analyses using an NJ tree also supported these findings, separating the two sheep breeds into distinct groups corresponding to their breed identities, thus reinforcing the genetic differentiation observed through PCA and ADMIXTURE analyses (Figure 3).

### 3.2 Genomic Signatures of Selection in Awassi Sheep

#### 3.2.1 ROH Analysis

**FIGURE 4:**
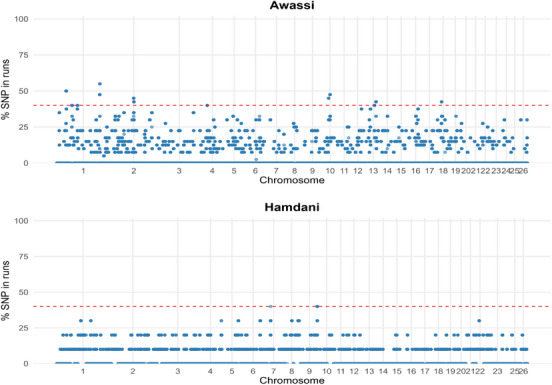
Manhattan plot of significant SNPs in ROH in Awassi and Hamdani sheep.

The ROH analysis in Awassi sheep identified 190 genes within regions of reduced genetic variation, indicative of historical selection pressures (Table S1). Of these, 22 genes emerged as key candidates with established associations with economically and biologically significant traits (Figure 4). Genes related to milk production and quality were prominent, including *CSN1S1*, *CSN1S2*, *CSN2* and *CSN3*, which encode casein proteins critical for milk protein content and cheese‐making properties. These findings align with the Awassi breed's reputation as a high‐yielding dairy sheep. Reproductive traits were also well‐represented, with *BMPR1B* (linked to the Booroola fecundity mutation and increased ovulation rate), *AANAT* (regulating melatonin synthesis and seasonal breeding), *CYP17A1* (steroid hormone synthesis), *PRL* (prolactin, influencing lactation and reproduction) and *TSHR* (thyroid‐stimulating hormone receptor, affecting metabolic and reproductive cycles) identified as candidate genes. Growth and meat quality traits were evident through *CAST* (calpastatin, regulating muscle tenderness), *FTO* (fat mass and obesity‐associated gene, impacting fat deposition), *GHR* (growth hormone receptor) and *MTOR* (mechanistic target of rapamycin, controlling muscle protein synthesis). Additionally, a strong signature of selection for disease resistance was observed, with genes such as *IL10, IL1A* and *IL1B* (interleukins that modulate inflammation), *MX1* and *MX2* (antiviral immunity), *SLC11A1* (involved in macrophage function) and *TLR2* (involved in pathogen recognition) detected in ROH regions. *SOD1* (superoxide dismutase 1) further suggests selection for oxidative stress resistance, enhancing resilience in arid environments. Finally, *ASIP* (agouti signalling protein) indicates selection for coat colour, a cultural and adaptive trait of significance.

In Hamdani sheep, the ROH analysis identified 277 genes within homozygous regions, with 30 being designated as key candidates associated with traits under historical selection (Figure 4) (Table S5). Meat quality and growth traits were strongly represented, with *CAST, CAPN1* and *CAPN3* (regulating muscle tenderness), *MSTN* (myostatin), *MYOD1* and *MYOG* (myogenic regulatory factors), *GH* (growth hormone) and *MTOR* (muscle protein synthesis) being identified as key factors. These genes highlight the breed's primary focus on meat production. Disease resistance was another primary signature, with immune‐related genes, including *CD14, CD247, CD28, CD3D, CD3E, CD4* (T‐cell function), *IFNAR1, IFNAR2, IFNG* (interferon signalling), *IL2, IL21, IL22, IL25, IL7* (interleukins), *MX1, MX2* (antiviral immunity), *SLAMF1, SLAMF9* (immune cell signalling) and *TLR3, TLR9* (pathogen recognition). *SOD1* (oxidative stress resistance) further supports resilience to environmental stressors. Reproductive traits were evident through *CYP17A1, HSD3B1* (steroidogenesis), *OVGP1* (fertilization), *POU1F1* (regulation of growth hormone and prolactin) and *TSHR* (involvement in metabolic and reproductive cycles). Genes involved in fat metabolism, such as *DGAT1* (milk fat synthesis), *LPL* (lipoprotein lipase), *PPARG* (adipogenesis) and *SCD* (fatty acid desaturation), suggest a selection for energy storage and meat quality. *ASIP* indicates historical selection for coat colour, whereas *VEGFA* (involved in angiogenesis) may relate to growth efficiency.

In the ROH analysis, Awassi sheep exhibited 20 discrete homozygosity islands spread across 10 chromosomes (2, 5, 10, 11, 12, 13, 17, 19, 23 and 26). These islands varied in length from approximately 0.67 Mb, as seen in the tract on chromosome 11, which spans from 9.14 to 9.81, to 1.37 Mb, exemplified by the region on chromosome 26, which ranges from 23.97 to 25.34 Mb (Table S1). In Hamdani sheep, we focused on four particularly prominent ROH regions on chromosomes 10, 11, 12 and 13, each spanning roughly 1.36 to 1.87 Mb: chromosome 10 (27.97–29.33 Mb, 1.36 Mb), chromosome 11 (21.81–23.55 Mb, 1.74 Mb), chromosome 12 (16.91–18.78 Mb, 1.87 Mb) and chromosome 13 (23.97–25.66 Mb, 1.69 Mb) (Table S5).

#### 3.2.2 iHS Analysis

**FIGURE 5:**
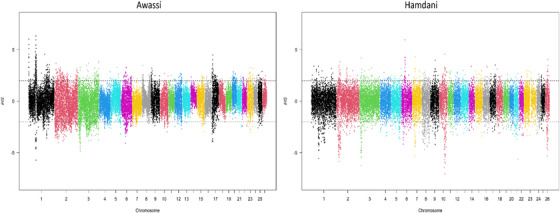
Genome‐wide distribution of iHS values for Awassi and Hamdani sheep across all autosomes.

The iHS analysis, which detects recent positive selection, identified 69 genes in Awassi sheep, with 19 emerging as significant candidates for traits under contemporary selective pressure (Figure 5) (Table S2). Disease resistance was a dominant signature, with *MX1, MX2* (antiviral immunity), *IFNAR1, IFNAR2* (interferon receptors), *IL12A* (Th1 immune response) and *SOD1* (oxidative stress resistance) showing extended haplotype homozygosity. These findings suggest that there is ongoing selection for health and survival in challenging climates. Genes associated with reproduction included *HSD3B1* (steroidogenesis), *OVGP1* (fertilization support) and *POU1F1* (a pituitary transcription factor that regulates prolactin and growth hormone). Growth and fat metabolism traits were represented by *ADIPOQ* (adiponectin, which regulates lipid metabolism), *CPT1B* (fatty acid oxidation), *GHSR* (growth hormone secretagogue receptor, involved in appetite regulation) and *POU1F1*. *SST* (somatostatin) further indicates the fine‐tuning of growth and metabolism. These results reflect recent selection pressures that align with the breed's dual‐purpose role in milk and meat production, as well as its adaptation to arid conditions.

The iHS analysis in Hamdani sheep identified 286 genes, with 34 highlighted as significant candidates under recent selection (Figure 5) (Table S4). Meat quality and growth traits were prominent, with *MSTN, MYF6* (muscle differentiation), *CAPN1, CAPN3, GH, GHSR* and *POU1F1* showing strong signals. Genes involved in fat metabolism, including *ADIPOQ, ADRB3* (lipolysis), *LPL, PPARG, FABP3* and *FABP4* (fatty acid transport), suggest ongoing selection for meat fat content and energy balance. Disease resistance was again a key focus, with *MX1, MX2, IFNAR1, IFNAR2, IFNG, IL12A, IL1A, IL1B, IL22, IL23A, IL7* (immune response), *SLC11A1* (macrophage function), and *TLR1, TLR6, TLR10* (pathogen recognition) detected. *SOD1* reinforces selection for stress resistance. Reproductive genes such as *BMPR1B* (ovulation rate), *CYP19* (oestrogen synthesis), *ESR2* (oestrogen receptor), *FSHR* (ovarian function), *HSD3B1, OVGP1* and *TSHR* suggest recent improvements in fertility. Milk production traits, although less dominant in Hamdani sheep, were represented by *ABCG2* (milk yield), *CSN1S1, CSN1S2, CSN2* and *CSN3* (milk protein), as well as *POU1F1*, indicating some selection for dairy potential. ASIP reflects continued selection for coat colour, aligning with breed characteristics.

The iHS scan (top 5% of iHS) in Awassi sheep revealed three candidate selective sweeps: a 1.00 Mb sweep on chromosome 1 (0.00–1.00 Mb), a 5.00 Mb sweep on chromosome 3 (190.00–195.00 Mb) and a 0.50 Mb sweep on chromosome 17 (4.00–4.50 Mb) (Table S2). In Hamdani sheep, four iHS regions of approximately 0.50 Mb each were detected on chromosomes 10 (52.00–52.50 Mb), 1 (14.20–14.70 Mb), 21 (42.15–42.65 Mb) and 26 (18.00–18.50 Mb) (Table S4).

### 3.3 Selection Signatures Based on Tajima's D

**FIGURE 6:**
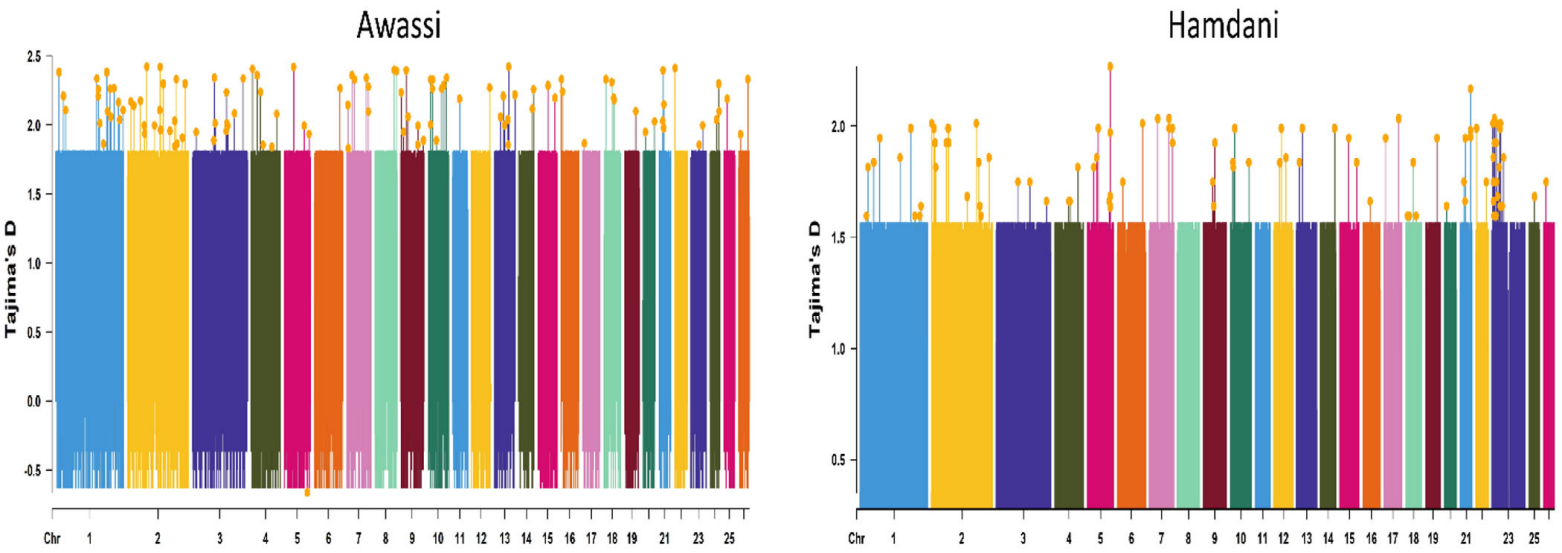
Genome‐wide distribution of Tajima's D values for Awassi and Hamdani sheep across all autosomes.

Tajima's D analysis, which detects deviations in allele frequency spectra suggestive of selection, identified a single candidate gene in Awassi sheep: *FGF12* (fibroblast growth factor 12) (Figure 6) (Table S3). This gene is associated with cellular signalling and development, potentially influencing growth or neurological function, though its specific role in sheep traits remains less defined compared to other candidates. The limited number of genes detected by Tajima's D suggests that this method may capture fewer signatures of selection in Awassi sheep compared to ROH and iHS, possibly due to weaker or more localized selective pressures detectable by this metric.

Tajima's D analysis in Hamdani sheep identified four candidate genes under potential selection: *CAST, PSMA6, MAPK14* and *CYB5A* (Figure 6) (Table S6). *CAST* (calpastatin) is a well‐established regulator of muscle tenderness, strongly corroborating its detection by ROH and iHS as a key gene for meat quality. *MAPK14* (mitogen‐activated protein kinase 14, also known as p38 MAPK) is involved in cellular stress responses and inflammation, potentially contributing to environmental adaptation and immune function. *CYB5A* (cytochrome b5 type A) plays a role in fatty acid desaturation and steroid metabolism, suggesting a link to meat fat composition or reproduction. *PSMA6* (proteasome subunit alpha 6) is involved in protein degradation, but its specific trait association in sheep remains less clear, potentially relating to cellular maintenance or growth. The detection of multiple genes by Tajima's D in Hamdani sheep, compared to only one in Awassi, may indicate a broader or more recent selective sweep in this breed.

Tajima's D analysis in Awassi sheep identified six windows departing significantly from neutrality, each window spanning between 0.10 and 0.41 Mb on chromosomes 1, 3 and 15—for example, a 0.41 Mb window on chromosome 3 (190.00–190.41 Mb) and a 0.32 Mb window on chromosome 1 (178.82–179.14 Mb) (Table S3). In Hamdani sheep, four significant Tajima's D windows were found on chromosomes 1 and 2, ranging from 0.01 Mb (chromosome 2: 1.84–1.85 Mb) to 0.24 Mb (chromosome 2: 10.78–11.02 Mb), none of which overlapped annotated genes (Table S6).

### 3.4 Identification of Selection Signatures

**FIGURE 7:**
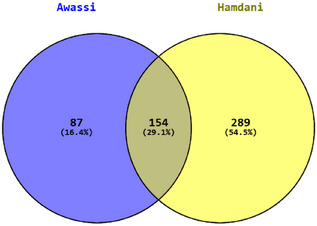
Venn diagram showing the overlap of genes identified by ROH, iHS and Tajima's D analyses in Awassi and Hamdani sheep.

We applied ROH, iHS and Tajima's D analyses in Awassi and Hamdani sheep to detect genomic regions under positive selection. As illustrated in the Venn diagram (Figure 7), 87 genes (16.4%) were uniquely identified in Awassi, 289 genes (54.5%) were unique to Hamdani, and 154 genes (29.1%) were common to both breeds. This overlap indicates the presence of breed‐specific selection signatures and shared genetic components. Notably, several common genes are involved in key biological processes, including growth, reproduction, immune response and adaptation. Among the common genes, for example, are *BMPR1B, BMP4, BMPR2, CAST, CFTR, IGFBP5, IL1A, IL1B, ASIP, FOXO3, TSHR, PRKAG3, ADIPOQ, SOD1* and *MX1*.

### 3.5 Functional Enrichment of Candidate Genes

**FIGURE 8:**
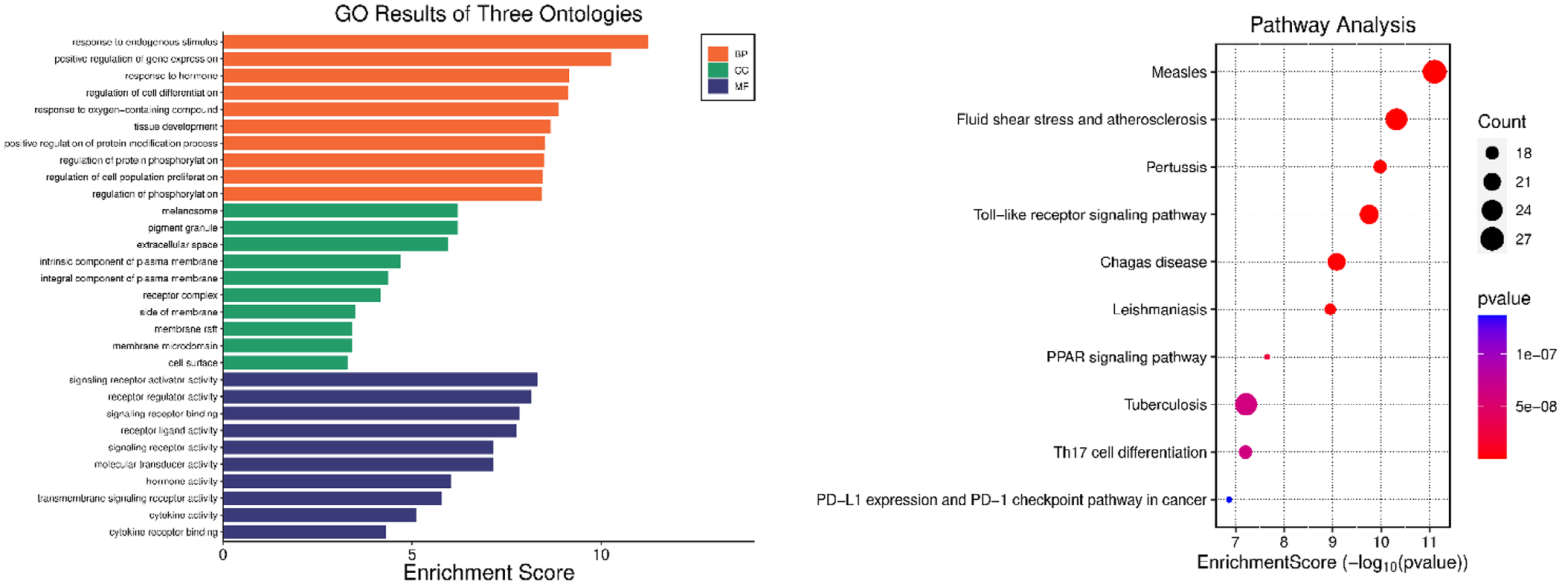
GO enrichment and pathway analysis of the identified genes, showing their involvement in various biological processes and signalling pathways.

Candidate genes within 100‐kb extended intervals around significant selection signals were identified through iHS, ROH and Tajima's D methods. GO and KEGG pathway enrichment analyses were performed to explore the biological functions of these candidate genes. The GO enrichment analysis categorized candidate genes into three main groups: BP, CC and MF. In the biological processes category, significant enrichment was observed in regulating cell proliferation, response to hormones, response to endogenous stimuli, positive regulation of gene expression, response to oxygen‐containing compounds and response to endogenous stimuli (Figure 8). Within the CC category, significant terms included cell surface, membrane microdomain, membrane raft, membrane side, receptor complex and extracellular space. The MF enrichment identified substantial associations with cytokine receptor binding, cytokine activity, hormone activity, signalling receptor activity and receptor–ligand activity. KEGG pathway analysis revealed candidate genes significantly enriched in multiple biologically relevant pathways, including the PD‐L1 expression and PD‐1 checkpoint pathway in cancer, Th17 cell differentiation, tuberculosis, PPAR signalling pathway, Toll‐like receptor signalling pathway, fluid shear stress and various immune and inflammatory pathways such as Th17 cell differentiation and Toll‐like receptor signalling. These enriched pathways suggest key roles for these candidate genes in immune response, adaptation and potentially disease resistance in Awassi and Hamdani sheep breeds.

## 4 Discussion

Our analysis, which incorporated PCA, ADMIXTURE and phylogenetic tree construction, unequivocally delineated the Awassi and Hamdani breeds as distinct genetic clusters. Individuals were segregated predominantly by breed, with Awassi and Hamdani forming non‐overlapping clusters along the PC axes. This segregation substantiates the hypothesis that these breeds represent distinct gene pools with limited recent gene flow, aligning with their historical recognition as separate breeds maintained within traditional breeding systems. The ADMIXTURE analysis, conducted at the optimal number of clusters (K), allocated nearly all Awassi individuals to a single ancestral component and Hamdani individuals to another, with negligible admixture observed. This finding indicates that crossbreeding between these breeds has been minimal. These results are consistent with previous mitochondrial DNA investigations, which revealed divergent maternal lineage signatures characterized by breed‐specific haplotypes in the 12S rRNA region (Fadhil and Al‐Shuhaib 2022). Our genome‐wide approach extends these observations beyond mitochondrial markers, demonstrating pervasive genomic divergence between the breeds. Despite the clear genetic differentiation between Awassi and Hamdani, the overall genomic divergence between these breeds remains moderate when compared to the spectrum of divergence observed across domestic sheep breeds (Kijas et al. 2012). Large‐scale analyses of global sheep populations have shown that breed‐level divergences typically cluster within intermediate differentiation ranges, reflecting both shared ancestry and recent selective pressures (Kijas et al. 2012; Wanjala et al. 2025). Similarly, moderate levels of genetic diversity and population structure have been reported across diverse sheep breeds in Europe and Asia, indicating that extreme divergences are uncommon at the breed level (Xiong et al. 2020; Saravanan et al. 2021). These patterns contrast sharply with interspecific genomic divergences within the genus *Ovis*, where comparisons between domestic sheep and their wild relatives regularly exceed high differentiation thresholds (e.g., X‐chromosomal variability analyses; global genome‐wide surveys) (Kijas et al. 2012; Chen et al. 2018). In light of these comparative frameworks, we clarify that although Awassi and Hamdani are genetically distinct breeds, their overall genomic divergence aligns with a moderate level of differentiation typical of many global sheep breeds (Kijas et al. 2012; Rodrigues et al. 2025). This pattern reflects the generally shallow population structure among sheep breeds, a consequence of sheep domestication from a broad genetic base. Even distinct breeds continue to share substantial ancestral polymorphism due to historical admixture events and large effective population sizes. Multiple genome‐wide studies show that Awassi and Hamdani sheep cluster within a single ancestral West Asian gene pool—thought to coincide with the primary centre of sheep domestication—from which they later diverged through region‐specific selection and breeding (Li, Yang et al. 2020; Brake et al. 2021; Fadhil and Al‐Shuhaib 2022; Lv et al. 2022). Although divergent selection is apparent from distinct selection signatures, neither breed exhibits signs of severe bottlenecks or prolonged isolation. Both retain high levels of within‐population genetic diversity, consistent with observations that sheep breeds generally maintain greater heterozygosity compared to specialized cattle or dog breeds (Kijas et al. 2012). Such genetic diversity is crucial for adaptability and future breeding efforts, as further supported by the absence of significant inbreeding. Additionally, although Awassi and Hamdani cluster with other Middle Eastern fat‐tailed sheep, they remain genetically distinct in broader phylogenetic analyses. For instance, in the global NJ tree reported by Li, Yang et al. (2020), both breeds were situated within the West Asian clade yet occupied distinct branches, reflecting their unique breed status. Our NJ tree of Iraqi sheep similarly demonstrates that Awassi samples cluster with other Levantine fat‐tailed breeds. In contrast, Hamdani samples form a separate cluster, exhibiting a closer affinity to breeds from Northern Iraq. This pattern corroborates the known geographical distribution, with Awassi being widespread across the Fertile Crescent (Iraq, Syria, Turkey) and Hamdani being more confined to Northern Iraq and Southeastern Turkey, thus facilitating subtle genetic drift. Supporting these findings, a microsatellite‐based study in Northern Iraq (Al‐Barzinji et al. 2011) also identified Hamdani as forming a genetically distinct group from other local breeds, reinforcing our genome‐wide SNP conclusions. In summary, the population structure analyses strongly indicate that breed management and historical selection have effectively preserved the genetic integrity of the Awassi and Hamdani breeds. The lack of recent admixture suggests that improvements have primarily been achieved through intra‐breed selection rather than crossbreeding, thereby affirming that the detected selection signals are indeed breed‐specific rather than artefacts of mixed ancestry. In the current study, ROH analysis in Awassi sheep identified 190 genes, of which 22 were deemed particularly significant. Notably, several of these genes belong to the casein family (*CSN1S1, CSN1S2, CSN2, CSN3*), which are directly involved in milk synthesis and quality. The prominence of these casein genes supports the established reputation of the Awassi breed as a high‐yielding dairy sheep, in agreement with previous studies that have documented the pivotal role of casein proteins in dairy production (Dixit et al. 2020; Li et al. 2022; Rezvannejad et al. 2022). In contrast, the ROH analysis in Hamdani sheep identified 277 genes, including 30 key candidates that are predominantly associated with meat quality and growth traits, such as *CAST, MSTN* and members of the calpain family (*CAPN1* and *CAPN3*). The detection of these genes corroborates the breed's specialization in meat production. Additionally, the identification of several immune‐related genes suggests that historical selection pressures in Hamdani sheep have favoured alleles that enhance disease resistance, a trait vital for survival under challenging environmental conditions (Purfield et al. 2017; Gebreselassie et al. 2019; Rodrigues et al. 2025). A comparative evaluation of the ROH results indicates that both breeds exhibit distinct signatures of historical selection, albeit with differing targets. In Awassi sheep, selection has predominantly favoured dairy traits, whereas in Hamdani sheep, the historical emphasis is on muscle development and immune response. The presence of shared genes, such as *SOD1* and *ASIP*, between the two breeds suggests that they have undergone common selective pressures—including adaptation to environmental stressors and regulation of coat colour—albeit with varying degrees of emphasis. These observations are consistent with earlier findings that link breed‐specific selection to distinct production goals (Norris and Whan 2008; Fariello et al. 2014; Yurchenko et al. 2019; Kalds et al. 2022). Moreover, numerous livestock studies have validated the ROH approach as an effective method for uncovering long‐standing selection events and inbreeding effects. For example, strong ROH signals in regions containing genes such as *BMPR1B*—implicated in reproductive efficiency—underscore the importance of reproductive traits in dairy breeds and corroborate previous reports associating this gene with enhanced ovulation rates (Bai et al. 2024; Bao et al. 2024; Zhao et al. 2024; Zhong et al. 2024). In addition to ROH analysis, the iHS method was employed to detect recent positive selection via extended haplotype homozygosity around core alleles. In Awassi sheep, iHS analysis identified 69 genes, of which 19 emerged as significant candidates. These genes include those involved in disease resistance (*MX1, MX2*) and metabolic efficiency (*ADIPOQ, CPT1B*), suggesting that recent selection has targeted traits related to health and energy balance. The application of iHS, for example, has been transformative in revealing recent adaptations in response to modern breeding practices and changing environmental conditions (Voight et al. 2006). Our identification of metabolic and immune‐related genes through iHS demonstrates how contemporary selection pressures are now captured with unprecedented resolution. Moreover, integrating multiple analytical approaches provides a holistic view of the genomic landscape, validating candidate genes and prioritizing those most likely to be functionally relevant—a critical step in moving from association to causation in genomic research (Nielsen 2005). The efficacy of the iHS method in detecting recent adaptive events has been well documented in studies across both human and livestock genomes (Cilloniz et al. 2012; Fadhil and Mercan 2016; Clark et al. 2017; Tosic et al. 2018; Li et al. 2021; Mohammed et al. 2022). In Hamdani sheep, the iHS analysis was even more expansive, revealing 286 genes with 34 candidates exhibiting strong signals of recent selection. The gene signatures in this breed further emphasize selection for meat quality and growth‐related traits, with notable candidates, including *MSTN, MYF6 and TLR1*. The recurrent identification of muscle development regulators, such as *MSTN*, aligns with previous research demonstrating that myostatin mutations are associated with muscle hypertrophy in various livestock species (Grobet et al. 1997; Rochus et al. 2018; Han et al. 2022). Furthermore, the prominent representation of genes related to fat metabolism (*ADIPOQ, ADRB3, LPL, PPARG*) in the iHS results for Hamdani sheep suggests that contemporary breeding practices may be fine‐tuning traits that affect meat composition and energy storage (McKenzie et al. 2010; Fariello et al. 2014; Liu et al. 2015; Deng et al. 2019; Li, Lu et al. 2020; Xu et al. 2023; Qi et al. 2024). The iHS results highlight the dual roles of selection, revealing that although both breeds exhibit signatures associated with disease resistance, the specific traits under recent selection differ by their respective production focuses. In Awassi sheep, for example, disease resistance appears to be driven by genes such as *IFNAR1* and *IFNAR2*, which play key roles in the antiviral response. Conversely, Hamdani sheep display a broader array of immune‐related genes, including *TLR1* and *TLR10*, which may contribute to enhanced pathogen recognition. These differences highlight the intricate interplay between production traits and health‐related selection, aligning with earlier studies that have reported similar breed‐specific patterns in other livestock species (Chang et al. 2009; Jann et al. 2009; Davies et al. 2022; Rodrigues et al. 2025). Tajima's D analysis provides a complementary perspective by identifying deviations in the allele frequency spectrum that may indicate balancing or directional selection. In Awassi sheep, this analysis identified a single candidate gene, *FGF12*. Although the role of *FGF12* in sheep production traits is not yet fully defined, its involvement in cellular signalling and development suggests that even isolated signals can reflect emerging selection pressures (Dzomba et al. 2023). The limited signal in Awassi sheep may be due to the sensitivity of Tajima's D to demographic changes, capturing only those sweeps that produce broad‐scale shifts in allele frequencies (Nielsen 2005). In contrast, Hamdani sheep exhibited four candidate genes (*CAST, PSMA6, MAPK14* and *CYB5A*) via Tajima's D. The recurrent detection of *CAST* across analyses strongly corroborates its role in meat tenderness, whereas *MAPK14*, a gene implicated in stress response and inflammation, suggests that environmental adaptation plays a critical role in this breed. Although *PSMA6* and *CYB5A* are less directly linked to production traits, their identification may indicate broader genomic changes related to cellular maintenance and metabolic regulation. These findings support previous reports on the sensitivity of Tajima's D to recent demographic events and subtle selection pressures (Akey 2009). The comparative differences in Tajima's D signals between Awassi and Hamdani sheep likely reflect breed‐specific historical dynamics. Awassi sheep appear to have undergone long‐term selection for dairy production, resulting in a relatively stable allele frequency distribution. In contrast, Hamdani sheep may have undergone more recent or more intense selective sweeps that affected a broader range of genes. This observation aligns with the notion that divergent production goals and environmental challenges can lead to markedly different selection landscapes (Nielsen 2005; Kijas et al. 2012; Fariello et al. 2014). The convergence of results from the ROH, iHS and Tajima's D methodologies further reinforces the robustness of these findings. For instance, the repeated detection of genes such as BMPR1B, SOD1 and ASIP in both breeds highlights common pathways underlying critical adaptive traits. BMPR1B, known for its association with reproductive efficiency (Zhao et al. 2024), was detected in Awassi sheep through ROH analysis and is similarly reflected in the selection signals observed in Hamdani sheep, albeit with a stronger emphasis on meat production. Likewise, the consistent appearance of *SOD1* across analyses underscores its role in oxidative stress resistance, a trait that is particularly important in arid and challenging environments (Yurchenko et al. 2019). The exclusive identification of casein genes (*CSN1S1, CSN1S2, CSN2, CSN3*) in Awassi sheep via ROH analysis further supports previous studies linking these gene clusters to milk quality in dairy sheep (Dixit et al. 2020; Rezvannejad et al. 2022). In contrast, the detection of muscle development genes (*MSTN, MYOD1, CAPN1*) in Hamdani sheep aligns with research demonstrating the critical role of these genes in meat quality and muscle accretion (Grobet et al. 1997; Fadhil and Zülkadir 2021). Notably, the differential Tajima's D signals between the two breeds warrant further investigation. Although Awassi sheep exhibit a single signal (*FGF12*), the broader range of genes identified in Hamdani sheep suggests a more dynamic or multifaceted selection process in the latter. This observation aligns with previous literature, which indicates that Tajima's D is particularly sensitive in populations that have undergone recent expansions or bottlenecks (Akey 2009). In summary, the methodological differences among ROH, iHS and Tajima's D highlight the importance of employing multiple approaches to obtain a comprehensive understanding of selection dynamics. Each method has inherent strengths and limitations. ROH analysis is effective for detecting long‐standing selection events and inbreeding effects, particularly in breeds that have experienced extensive artificial selection. In contrast, the iHS method is adept at capturing recent selection but may yield false positives in populations with complex demographic histories (Voight et al. 2006). Finally, Tajima's D remains a robust statistical tool for detecting deviations in allele frequency spectra; however, its sensitivity may be limited when selection shifts are subtle or obscured by demographic events. The complementary nature of our methods is evident in the convergence of candidate genes, such as *CAST* in Hamdani sheep and *BMPR1B* in Awassi sheep, across multiple analytical approaches, which enhances confidence in the biological relevance of these signals. Conversely, the discrepancy observed with Tajima's D in Awassi sheep—where only *FGF12* was detected—highlights the importance of relying on multiple methods to avoid overlooking specific selection signals. This finding highlights the importance of utilizing a comprehensive suite of genomic tools to capture the full spectrum of selection dynamics, as emphasized in the broader livestock genomics literature (Nielsen 2005; Kijas et al. 2012). The divergence in selection signatures between Awassi and Hamdani sheep reflects their distinct production purposes. Awassi sheep have historically been selected for dairy traits, as demonstrated by the enrichment of genes associated with milk protein synthesis (e.g., casein genes) and reproductive efficiency (e.g., *BMPR1B*). This dairy specialization is further supported by iHS findings, which reveal strong signals in genes linked to metabolic efficiency and disease resistance—traits crucial for maintaining high milk yields under variable environmental conditions (Li et al. 2022). These patterns align with previous reports that have characterized Awassi sheep as a breed evolving under intense selection for lactation, with an underlying genetic architecture optimized for milk production (Kijas et al. 2012). In contrast, Hamdani sheep have been predominantly selected for meat quality and growth. Both ROH and iHS analyses consistently highlight genes involved in muscle development (e.g., *MSTN* and *MYOD1*) and meat tenderness (e.g., *CAST* and *CAPN* genes). Additionally, the detection of various immune‐related genes suggests a dual role in enhancing production efficiency and disease resistance. This dual selection pressure likely reflects a balance between optimizing meat yield and ensuring resilience against environmental challenges, which is consistent with findings in other meat‐specialized breeds (Grobet et al. 1997; Fariello et al. 2014). A noteworthy observation is the presence of overlapping genes between the two breeds. For instance, the presence of *SOD1* in both Awassi and Hamdani sheep suggests that oxidative stress resistance is a universally advantageous trait, regardless of the primary production objective. Similarly, the recurrent detection of immune‐related genes—such as *MX1* and various members of the *TLR* family—indicates common selection pressures imposed by pathogens and harsh environmental conditions. These shared signals suggest that although directional selection for production traits may differ, there is a fundamental requirement for robust disease resistance and environmental adaptability across diverse sheep breeds. Our results resonate with seminal studies in livestock genomics. Early work by Kijas et al. (2012) demonstrated that a combination of historical admixture and selection for specific production traits has shaped the genomic architecture of sheep breeds, forming distinct clusters corresponding to dairy and meat types—a finding strongly supported by our data. Likewise, Fariello et al. (2014) highlighted the value of integrating diverse analytical methods for detecting selection signatures, a strategy that our study also adopts robustly. Recent advancements in whole‐genome sequencing and SNP chip technologies have enabled the detection of subtle selection signals that were previously elusive. Functional enrichment analyses further corroborated our findings, highlighting gene categories predominantly associated with growth, immune response, reproduction and adaptation. Genes involved in immune and inflammatory pathways, such as Toll‐like receptor signalling (Olech et al. 2021), PPAR signalling (Liu et al. 2022) and Th17 differentiation, underscore significant adaptive responses to environmental stressors, pathogen challenges and inflammatory conditions (Li et al. 2024). The enrichment of pathways related to *PD‐L1* expression, *PD‐1* checkpoint regulation (Tiyamanee et al. 2023) and cytokine signalling further emphasizes complex immune modulation mechanisms critical for breed adaptability and resilience (Abendaño et al. 2021; Olech et al. 2021). Additionally, candidate genes were significantly associated with biological processes, including hormonal responses, which may influence reproductive cycles, lactation and growth rates. The identification of enriched terms, such as regulation of cell proliferation, further highlights the genetic basis for enhanced growth and tissue development, which are essential for productivity in both breeds (Wang et al. 2020; Xi et al. 2024). Localization of these candidate genes within membrane‐associated structures—such as membrane rafts, receptor complexes and extracellular spaces—underscores their vital roles in cellular signalling and environmental responsiveness (Peng et al. 2022). Collectively, these analyses elucidate the multifaceted genetic adaptations that have shaped the Awassi and Hamdani breeds. The identification of candidate genes and pathways with considerable potential for enhancing disease resilience, reproductive efficiency and overall productivity provides a valuable foundation for future selective breeding programmes.

## 5 Conclusion

In this study, we investigated population structure and selection signatures in two important Iraqi sheep breeds, Awassi and Hamdani, using genome‐wide SNP data generated by the Illumina Ovine SNP50K chip. Our analyses revealed clear genetic differentiation between the breeds, reflecting their distinct genetic histories and breeding practices. Using three complementary approaches (iHS, ROH and Tajima's D), we identified candidate genomic regions and key genes that are potentially under positive selection, many of which are associated with economically relevant traits, including growth, milk production, reproduction, immunity and adaptation. These findings provide valuable insights into breed‐specific selection pressures and genetic resources, highlighting potential targets for breeding and conservation programmes to improve sheep productivity and adaptability in Iraq.

## Author Contributions

**Mervan Bayraktar**: Conceptualization, methodology, formal analysis, investigation, visualization, writing – original draft. **Omer Shoshin**: Data collection, laboratory analysis and visualization. **K. A. Saravanan**: Review and editing, data analysis, visualization. **Tahreer M. Al‐Thuwaini**: Review and editing. **Gökhan Gökçe**: Review and editing. **Zeynab Hussein Fadhil**: Writing – review and editing, visualization. **Melis Çelik Güney**: Visualization.

## Conflicts of Interest

The authors declare no conflicts of interest.

## Peer Review

The peer review history for this article is available at https://www.webofscience.com/api/gateway/wos/peer‐review/10.1002/vms3.70584.

## Supporting information




**Table S1**. Signatures of selection detected by ROH in Awassi.

**Table S2**. Signatures of selection detected by iHS in Awassi.

**Table S3**. Signatures of selection detected by Tajima's D in Awassi.

**Table S4**. Signatures of selection detected by iHS in Hamdani.

**Table S5**. Signatures of selection detected by ROH in Hamdani.

**Table S6**. Signatures of selection detected by Tajima's D in Hamdani.

## Data Availability

Data are available from the corresponding author upon reasonable request.
